# Draft Genome Sequences of Four *Dickeya dianthicola* and Four *Dickeya solani* Strains

**DOI:** 10.1128/genomeA.00087-12

**Published:** 2013-07-25

**Authors:** Leighton Pritchard, Sonia Humphris, Steve Baeyen, Martine Maes, Johan Van Vaerenbergh, John Elphinstone, Gerry Saddler, Ian Toth

**Affiliations:** Information and Computational Sciences (ICS), James Hutton Institute, Invergowrie, Dundee, Scotland, United Kingdoma; Cellular and Molecular Sciences (CMS), James Hutton Institute, Invergowrie, Dundee, Scotland, United Kingdomb; ILVO, Merelbeke, Belgiumc; Food and Environment Research Agency (FERA), Sand Hutton, York, United Kingdomd; Science and Advice for Scottish Agriculture (SASA), Roddinglaw Road, Edinburgh, United Kingdome

## Abstract

*Dickeya dianthicola* and “*Dickeya solani*” are currently the dominant bacterial pathogens of potatoes in Europe. Here, we present the draft genome sequences of four strains of each pathogen.

## GENOME ANNOUNCEMENT

Enterobacterial plant pathogens belonging to the *Dickeya* genus (previously *Erwinia chrysanthemi*) cause disease worldwide in a wide range of plant species, including crops (1). The *Dickeya* genus is currently considered to comprise 5 species: *Dickeya dianthicola*, *Dickeya dadantii*, *Dickeya zeae*, *Dickeya chrysanthemi*, and *Dickeya paradisiaca* (2, 3). *D. dianthicola*, although causing disease worldwide on a range of crops and ornamentals, has had great impact on potato production in Europe from the early 1970s onward (1). In addition to the five assigned *Dickeya* species, several isolates were identified that formed distinct clades, e.g., DUC-1 (also referred to as group 1, clade IV), DUC-2, DUC-3, SLC-1, and SLC-2, which may represent previously unidentified species. Since 2004, DUC-1, for which the name “*Dickeya solani*” has been proposed, has been identified across Europe as causing disease in potatoes, and it was also isolated recently from hyacinth (4–8).

Currently, there are four complete sequenced *Dickeya* strains deposited in GenBank, one each from *D. paradisiaca*, *D. zeae*, *D. chrysanthemi*, and *D. dadantii*, as well as a draft *D. zeae* strain. Here, we announce draft genome sequences of the following eight strains from previously unsequenced *Dickeya* species: *D. dianthicola* NCPPB 453^T^, a type strain from *Dianthus*; NCPPB 3534, from potato in the Netherlands; IPO 980, from potato in the Netherlands; LMG 25864, from potato in Belgium; and *D. solani* strain IPO 2222 from potato in the Netherlands; MK10, from potato in Israel; MK16, from river water in the United Kingdom; and LMG 25865, from potato in Belgium.

Six strains were sequenced using 454 pyrosequencing (Roche, Branford, CT): IPO 2222, MK10, MK16, NCPPB 453^T^, NCPPB 3534, and IPO 980. Three strains were sequenced using Illumina GAIIx: LMG 25864, LMG 25865, and NCPPB 3534. Three strains were assembled *de novo* using 454 Life Sciences Newbler v2.5.3 (IPO 2222, NCPPB 453^T^, and IPO 980); strain LMG25865 was assembled by reference mapping to the IPO 2222 assembly using CLC bio assembly module, strain NCPPB 3534 was assembled as a hybrid of 454 and Illumina reads using MIRA, strains MK10 and MK16 were assembled as a meta-assembly of Newbler *de novo* and reference-guided assemblies to the IPO 2222 assembly, and the LMG 25864 strain was assembled as a meta-assembly of CLC and Velvet *de novo* assemblies.

Sequences were annotated using a combination of Prodigal and RAST gene callers, BLAST searches using query sequences known to be missed by those packages, and tRNAScan-SE. A total of 4,571 (for strain LMG 25864), 4,477 (strain LMG 25865), 4,642 (strain NCPPB 3534), 4,365 (strain NCPPB 453^T^), 4,516 (strain IPO 980), 4,519 (strain MK10), 4,401 (strain MK16), and 4,471 (strain IPO 2222) genes were determined.

A detailed comparative genomic analysis of the eight draft sequences will follow in a future publication.

### Nucleotide sequence accession numbers.

The draft sequences of these eight *Dickeya* strains are available in GenBank under the accession no. AONU00000000 (IPO 2222), AOOP00000000 (MK10), AOOQ00000000 (MK16), AOOB00000000 (NCPPB 453^T^), AOOK00000000 (NCPPB 3534), AOOS00000000 (IPO 980), AOOM00000000 (LMG 25864), and AONX00000000 (LMG 25865). See Table 1 for statistics for the eight draft *Dickeya* genomes.

**TABLE 1:** Statistics for the eight draft *Dickeya* genomes

Species	Strain	Accession no.	No. of contigs	No. of assembled bases	N_50_
*D. dianthicola*	LMG 25864, GBBC 2039	AOOM00000000	237	4,767,435	35,250
*D. dianthicola*	NCPPB 3534	AOOK00000000	52	4,832,425	213,434
*D. dianthicola*	NCPPB 453^T^	AOOB00000000	47	4,668,129	235,227
*D. dianthicola*	IPO 980	AOOS00000000	63	4,825,313	181,825
*D. solani*	LMG 25865, GBBC 2040	AONX00000000	224	4,812,070	40,901
*D. solani*	MK10	AOOP00000000	39	4,930,219	295,556
*D. solani*	MK16	AOOQ00000000	23	4,867,774	485,700
*D. solani*	IPO 2222	AONU00000000	91	4,857,348	99,673
